# Pin1 Downregulation Is Involved in Excess Retinoic Acid-Induced Failure of Neural Tube Closure

**DOI:** 10.3390/ijms25115588

**Published:** 2024-05-21

**Authors:** Yuwen Chen, Jiao Pang, Lu Ye, Zhentao Zhang, Junfeng Kang, Zhuotao Qiu, Na Lin, Hekun Liu

**Affiliations:** Fujian Key Laboratory of Translational Research in Cancer and Neurodegenerative Diseases, The School of Basic Medical Sciences, Fujian Medical University, Fuzhou 350122, China; chenyuwen@fjmu.edu.cn (Y.C.); pj@fjmu.edu.cn (J.P.); yelu@fjmu.edu.cn (L.Y.); zzt@fjmu.edu.cn (Z.Z.); 2230130018@fjmu.edu.cn (J.K.); qzt@fjmu.edu.cn (Z.Q.); linna1088@fjmu.edu.cn (N.L.)

**Keywords:** Pin1, neural tube defect (NTD), Notch1, Nrf2, oxidative stress, endoplasmic reticulum stress

## Abstract

Neural tube defects (NTDs), which are caused by impaired embryonic neural tube closure, are one of the most serious and common birth defects. Peptidyl-prolyl cis/trans isomerase 1 (Pin1) is a prolyl isomerase that uniquely regulates cell signaling by manipulating protein conformation following phosphorylation, although its involvement in neuronal development remains unknown. In this study, we explored the involvement of Pin1 in NTDs and its potential mechanisms both in vitro and in vivo. The levels of Pin1 expression were reduced in NTD models induced by all-trans retinoic acid (Atra). Pin1 plays a significant role in regulating the apoptosis, proliferation, differentiation, and migration of neurons. Moreover, Pin1 knockdown significantly was found to exacerbate oxidative stress (OS) and endoplasmic reticulum stress (ERs) in neuronal cells. Further studies showed that the Notch1-Nrf2 signaling pathway may participate in Pin1 regulation of NTDs, as evidenced by the inhibition and overexpression of the Notch1-Nrf2 pathway. In addition, immunofluorescence (IF), co-immunoprecipitation (Co-IP), and GST pull-down experiments also showed that Pin1 interacts directly with Notch1 and Nrf2. Thus, our study suggested that the knocking down of Pin1 promotes NTD progression by inhibiting the activation of the Notch1-Nrf2 signaling pathway, and it is possible that this effect is achieved by disrupting the interaction of Pin1 with Notch1 and Nrf2, affecting their proteostasis. Our research identified that the regulation of Pin1 by retinoic acid (RA) and its involvement in the development of NTDs through the Notch1-Nrf2 axis could enhance our comprehension of the mechanism behind RA-induced brain abnormalities.

## 1. Introduction

Neural tube defects (NTDs) are one of the most prevalent congenital anomalies that arise during brain development, significantly impacting the population’s health. They is caused by impaired embryonic neural tube closure [1]. As a severe central nervous system malformation disorder, their primary symptoms include anencephaly, encephalocele, meningocele, spina bifida/recessive spina bifida, and cleft lip and palate [2]. The cause of NTDs is multifaceted, and the mechanisms behind them are still unknown. Genetic, environmental, nutritional, and maternal factors are involved in the progression of NTDs. In China, there is a relatively high prevalence of fetal NTDs, with around 80,000–100,000 newborns affected by NTDs annually. The prevalence of NTDs in northern China stands at around 10‰, a figure noticeably higher than the global average of 0.5–2‰. This has placed a substantial strain on both the nation and society [3]. Therefore, studying susceptibility genes and their molecular mechanisms that inhibit the development of neural tube defects is crucial for the diagnosis and treatment of NTDs.

Retinoic acid (RA), as a metabolite of vitamin A, is intricately linked to brain development [4,5]. Both insufficient and an excessive amounts of RA can result in brain abnormalities. All-trans retinoic acid (Atra) is the isomeric form of retinoic acid (RA) that can bind to the retinoic acid receptor (RAR), thereby influencing the transcriptional activity that impacts the development of the nervous system. It has been shown that RAR knockout mice are susceptible to abnormalities in embryonic nervous system development [6]. Mutations in the RAR receptor gene were also found in patients with meningoencephalocele, suggesting that the function of the RAR is associated with the pathogenesis of meningoencephalocele [7]. Furthermore, the Atra-induced NTD model is recognized as a dependable model for investigating the mechanisms of NTDs during embryonic development due to its effectiveness as a teratogenic agent [8]. However, there has been limited research on the correlation between Atra-induced NTDs and the proliferation, differentiation, and migration of neural stem cells, along with the associated molecular pathways. As a Pin1 inhibitor, Atra can promote its degradation [9]. Given the correlation between Atra and Pin1, Atra may modulate Pin1 signaling in NTDs.

Peptidyl-prolyl cis/trans isomerase 1 (Pin1), which belongs to the prolyl cis/trans isomerase family, encodes a distinctive group of peptidyl-prolyl cis/trans isomerases (PPIases). Pin1 is a protein linked to NIMA and has the ability to control mitosis [10]. It is characterized by the phosphorylation of serine (Ser) or threonine (Thr) as an amino acid preceding proline and cis-trans isomerization of the phosphorylated Ser/Thr-Pro (pSer/Thr-Pro) amino acid sequence to combine directly with the substrate [11]. Pin1 possesses two protein domains, with one being its N-terminal WW domain. The WW domain of Pin1 usually interacts exclusively with the pSer/Thr-Pro motif, which serves as a crucial site for modulating the phosphorylation of Pin1 substrate proteins. The other domain is the C-terminal peptidyl-prolyl cis-trans isomerase (PPIase) domain, which is an isomer of peptidyl-prolyl in the substrate protein and is involved in phosphorylation, protein—protein interactions, subcellular localization, protein stability, and other physiological functions [11,12]. Many studies have shown that an imbalance in Pin1 expression is closely related to the development of many diseases, such as neurodegeneration, tumors, and asthma. Additionally, Pin1 has the potential to enhance the differentiation of neural stem cells by interacting with β-catenin. The expression of Pin1 is upregulated during brain development, with Pin1 knockout leading to the inhibition of neurogenesis [13]. However, the possible role of Pin1 in Atra-induced brain abnormalities is largely unknown.

The Notch1-Nrf2 signaling axis is closely related to cellular lipid peroxidation and ferroptosis processes, which are associated with the occurrence of NTDs. Among them, Nrf2 has an antioxidant protection effect, can reduce neuron damage, and delays disease progression [14]. The Notchl/Hes1 signaling pathway is capable of enhancing the proliferation and differentiation of hippocampal neural stem cells in rats with cerebral ischemia, leading to the improvement of neurological function. The Notch1-Nrf2 signaling axis is an important signal transduction pathway in oxidative stress. The interaction and crosstalk of Notch1 and Nrf2 participate in the occurrence and development of degenerative disease in the nervous system and tumors. At the same time, Notch1-Nrf2 may play a synergistic role in the antioxidant regulation of myogenic stem cells, helping to maintain the self-renewal ability of myogenic stem cells and other phenotypes [15]. However, there are few reports about the effect of the Notch1-Nrf2 signaling pathway on the antioxidation and anti-injury of neurons. The function of both Notch1 and Nrf2 is closely related to Pin1 [16,17], and whether Pin1 plays a role in neurodevelopmental processes through the Notch1-Nrf2 axis warrants further investigation.

In this study, mouse and zebrafish models of brain abnormalities were established by administering excess Atra, and the regulation of Pin1 expression was evaluated. Furthermore, the regulatory effect of Pin1 and the molecular mechanism of brain abnormalities caused by Atra treatment were identified. The results of this study indicate that Pin1 might play an important role in Atra-induced brain abnormalities. These results may provide a novel direction for the clinical prevention of brain abnormalities.

## 2. Results

### 2.1. Atra Causes Brain Abnormalities in Mouse and Zebrafish Embryos

**Figure 1:**
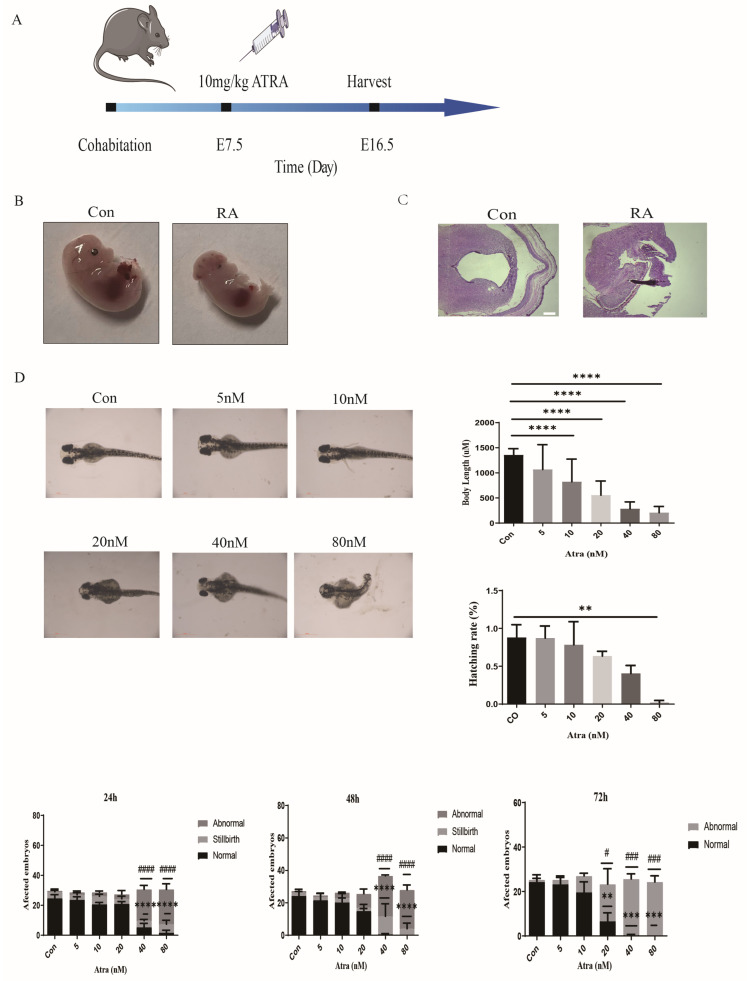
Illustration of the noticeable brain abnormalities induced by RA in mouse and zebrafish embryos. (**A**) A diagram illustrating the delivery of Atra to pregnant mice. (**B**) Images of 16.5-day-old pregnant mouse embryos obtained by bright-field microscopy from the control or Atra groups. (**C**) Hematoxylin and eosin staining of the brains of control-treated and Atra-treated embryos (scale bar = 400 μm). (**D**) Neural tube development in zebrafish embryos showed abnormalities when exposed to Atra from 4 to 72 hpf (scale bar = 500 μm). The data are shown as the means ± SDs from three independent experiments. ** *p* < 0.01, *** *p* < 0.001, and **** *p* < 0.0001 between the indicated groups. # *p* < 0.05, ### *p* < 0.001, and #### *p* < 0.0001 between the indicated groups.

Mice and zebrafish were chosen as the animal models for this study. To determine whether Atra affects embryo development, we first exposed mice to Atra, as shown in Figure 1A. Atra was administered to female mice on E7.5; then, their brains were examined using H&E staining for morphological alterations at E16.5 in both Atra-treated and control mice. The mice in the control group developed normally, with a complete appearance, normal and full skull development, and a ruddy color (Figure 1B). The Atra-treated embryos exhibited neural tube malformations, mainly cranial spina bifida deformities, which showed different degrees of developmental retardation and abnormal morphology; small embryo volume; and blood stasis (Figure 1B). H&E staining revealed that the embryonic nerve epithelium of the control group was well developed, the neural tube was closed, and the structure was complete (Figure 1C). Following Atra treatment, the embryonic neural tube did not close properly, resulting in a weak nerve epithelium with irregular arrangement, reduced cell numbers, disappearance of the marginal layer, structural defects, and incomplete closure of the top plate (Figure 1C). The phenotype of the zebrafish was analyzed after 72 h of exposure to Atra, and we found that after Atra exposure, the zebrafish embryos were obviously deformed, abnormally developed, and even dead. The telencephalon and diencephalon were diminished or even lost, the body axis became shorter, and the tail turned up (Figure 1D). Atra induces brain abnormalities in embryos of mice and zebrafish.

### 2.2. Pin1 Expression Was Reduced in Embryos and Neurons, Induced by RA and NTDs in Patients

**Figure 2:**
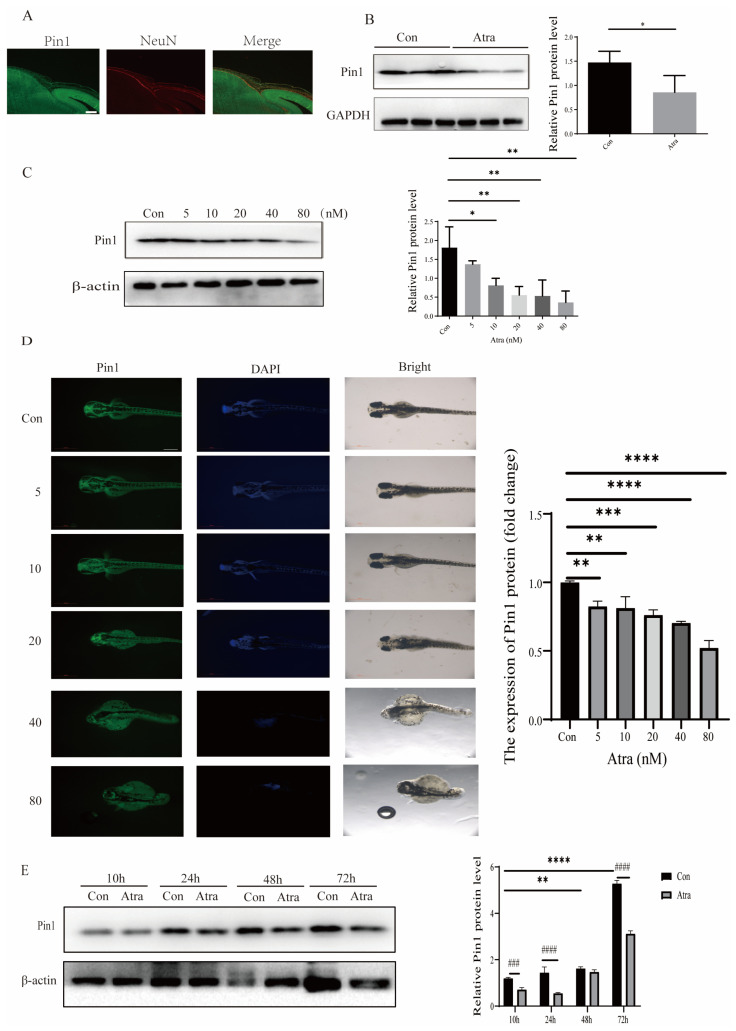
The immunolocalization and expression of Pin1 in embryos. (**A**) Pin1 was shown to be localized on the epithelial layers in control media (Con, green; scale bar = 50 μm). (**B**) Detection of Pin1 expression in E16.5 mouse brains treated with either control (Con) or Atra media using Western blot analysis. (**C**) Expression of Pin1 in zebrafish embryos cultured in control media (Con) or media supplemented with different doses of Atra, as detected via Western blotting. (**D**) Immunostaining was employed to identify Pin1 expression in zebrafish embryos. (**E**) Pin1 expression was assessed by Western blotting in zebrafish embryos cultured in control media (Con) or Atra media for various time periods (scale bar = 500 μm). The data are shown as the means ± SDs from three independent experiments. * *p* < 0.05, ** *p* < 0.01, *** *p* < 0.001, and **** *p* < 0.0001 between the indicated groups. ### *p* < 0.001, and #### *p* < 0.0001 between the indicated groups.

To investigate the impact of Atra-induced Pin1 events on brain development, we assessed Pin1 expression in a cell model of NTDs, as well as in brain samples from Atra-induced mice and zebrafish. Interestingly, Pin1 was downregulated in RA-treated neurons. Immunofluorescence staining analysis of mouse embryonic brain tissue showed that Pin1 expression is present in the neuroepithelium of embryonic mouse tissue (Figure 2A), suggesting a significant involvement of Pin1 in neural tube development. Moreover, compared with that in the control group, Pin1 protein expression in the embryonic brain tissue of Atra-treated NTD mice decreased significantly (Figure 2B). In zebrafish embryos exposed to varying concentrations of Atra, the trend was for Pin1 expression to decrease in a dose-dependent fashion (Figure 2C). Immunofluorescence was performed to assess Pin1 expression following exposure to varied concentrations of Atra. The levels of Pin1 expression decreased in correlation with increasing Atra concentration, mirroring the trends observed in the WB results (Figure 2D). To investigate the expression pattern of Pin1, we further detected Pin1 expression during the development of zebrafish embryos. From 10 hpf to 72 hpf, the expression of Pin1 in zebrafish embryos gradually increased with time, and Pin1 expression decreased after Atra treatment (Figure 2E), suggesting that Pin1 plays an essential role in the development of zebrafish embryos and that the teratogenic effect of Atra is related to a reduction in Pin1 expression. To further study the role of Pin1 in the occurrence of NTDs, NTDs and normal fetal brain tissue proteins were also collected for detection. Additionally, to confirm our findings, neural tissues of one fetus with NTDs and one control were collected. The expression of Pin1 protein in the neural tissue of the fetus with NTDs was notably lower compared to the normal control fetus (Supplementary Figure S1A), providing further confirmation of the pivotal role of Pin1 in the development of NTDs.

### 2.3. Pin1 Influenced the Apoptosis, Proliferation, Differentiation, and Migration of Neurons In Vitro

**Figure 3:**
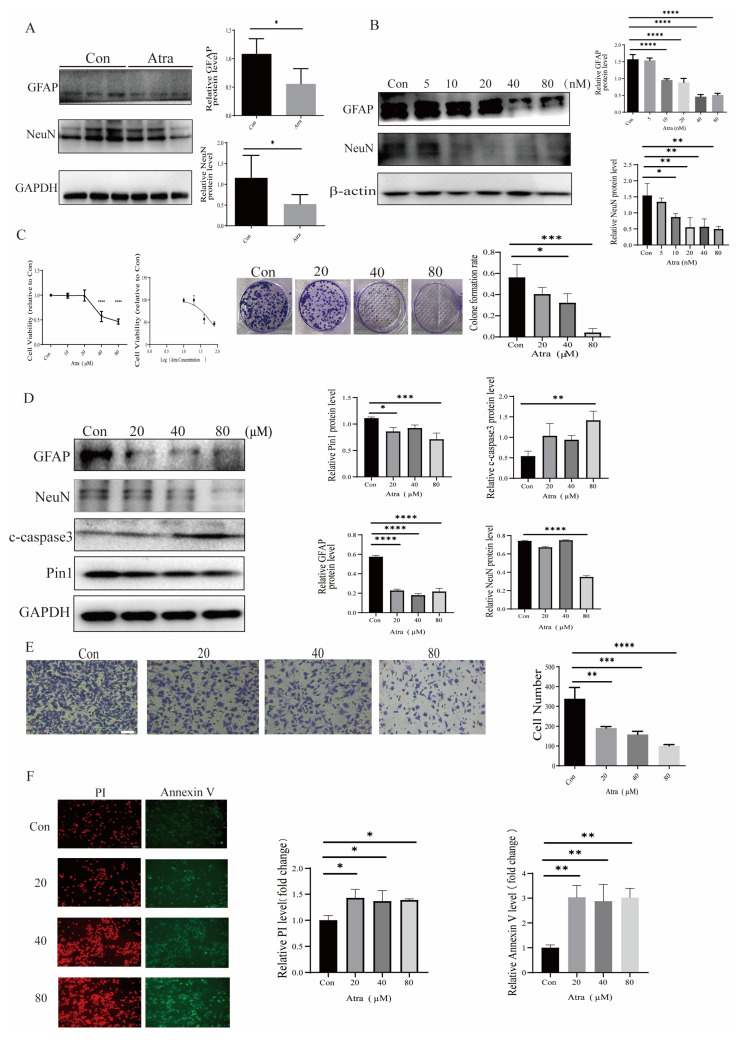
Atra treatment induced neuronal damage in a dose-dependent manner. Cells were exposed to different doses of Atra. (**A**) The levels of NeuN and GFAP in Atra-treated model mice were detected by WB. (**B**) The levels of NeuN and GFAP in Atra-treated zebrafish were detected by WB. (**C**) MTT and colony formation assays were used to assess the growth of SH-SY5Y cells. (**D**) The expression levels of NeuN, GFAP, and Pin1 in SH-SY5Y cells were determined using WB. (**E**) Transwell assays were carried out to analyze the migration ability of SH-SY5Y cells (scale bar = 100 μm). (**F**) PI and annexin V staining were utilized to evaluate the apoptosis of SH-SY5Y cells (scale bar = 100 μm). The data are shown as the means ± SDs from three independent experiments. * *p* < 0.05, ** *p* < 0.01, *** *p* < 0.001, and **** *p* < 0.0001 between the indicated groups.

**Figure 4:**
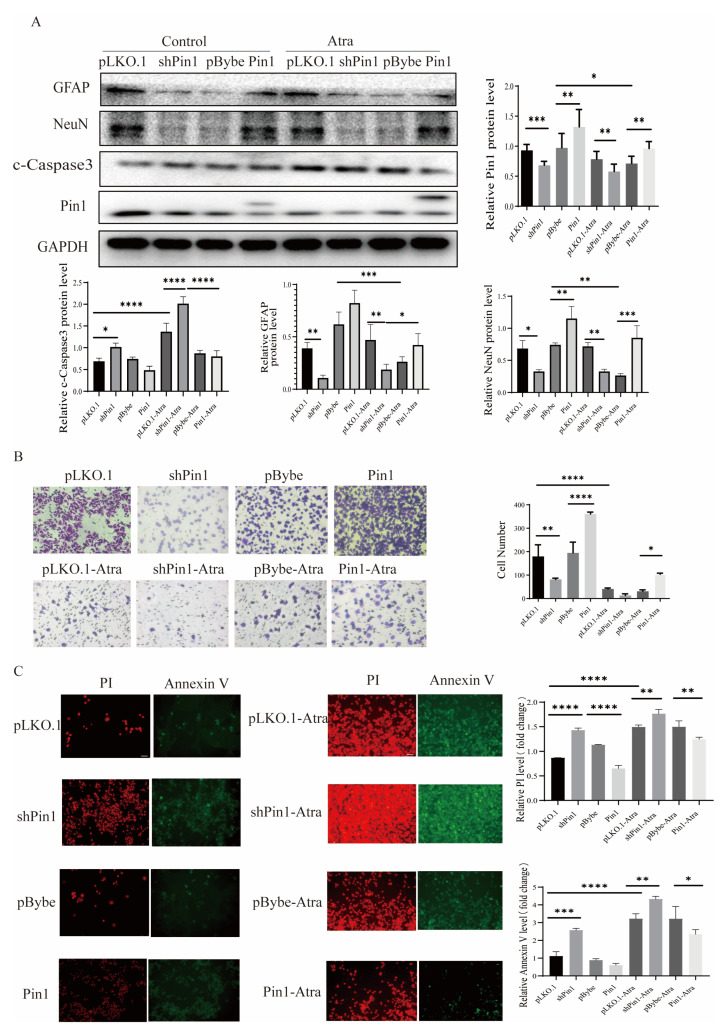
Pin1 affects neuron cell functions. Cells were infected with control, Pin1 shRNA, or Pin1. (**A**) The levels of NeuN, GFAP, c-caspase 3, and Pin1 in SH-SY5Y cells were detected by WB. (**B**) Transwell assays were carried out to analyze the migration ability of SH-SY5Y cells (scale bar = 100 μm). (**C**) PI and annexin V staining were utilized to evaluate the apoptosis of SH-SY5Y cells (scale bar = 100 μm). The data are shown as the means ± SDs from three independent experiments. * *p* < 0.05, ** *p* < 0.01, *** *p* < 0.001, and **** *p* < 0.0001 between the indicated groups.

The normal development of neural tubes relies on the proliferation, differentiation, and migration of neural stem cells (NSCs), which form the cytological foundation. Western blotting was used to detect the expression of neurodifferentiation-related indicators GFAP and NeuN. The expression of GFAP and NeuN decreased in the neural tissue of fetuses with NTDs compared to normal control fetuses (Supplementary Figure S1B), confirmed the pivotal role of differentiation in the development of NTDs. Then, the levels of GFAP and NeuN were determined in not only Atra-treated mouse and zebrafish models but also in in vitro cell models, and the results showed that the expression of GFAP and NeuN decreased with increases in Atra up to the teratogenic dose (Figure 3A,B,D), indicating that high concentrations of Atra affected the differentiation of neurons. MTT and colony formation assays demonstrated that Atra treatment significantly decreased the viability and suppressed the proliferation of SH-SY5Y cells in a dose-dependent manner, as shown in Figure 3C. Transwell migration experiments revealed that the migration capacity of cells decreased to different extents as the Atra treatment increased (Figure 3E). In addition, after Atra treatment, Pin1 protein levels decreased in SH-SY5Y cells, suggesting that Pin1 mediated Atra-induced neuronal damage in SH-SY5Y cells (Figure 3D). As the concentration of Atra increased, both PI and annexin V also increased, indicating a rise in the apoptosis rate of SH-SY5Y cells, as depicted in Figure 3F. To further explore the role of Pin1 in Atra-induced neuronal damage, we used lentiviral infection to construct transgenic SH-SY5Y cells. The migration ability was slightly reduced in the cell models with Pin1 knockdown, while Pin1 overexpression increased the migration ability (Figure 4B). Similarly, Pin1 promoted cell differentiation, and Pin1 knockdown inhibited SH-SY5Y cell differentiation. c-Caspase3 protein levels were used to investigate whether Atra exposure influenced apoptosis (Figure 3D and Figure 4A). An elevation in c-Caspase3 level was observed in cells treated with Atra. Knocking down Pin1 further heightened the levels of c-Caspase3, while overexpressing Pin1 reversed the Atra-induced increase in c-Caspase3 levels (Figure 4A). These findings were in line with the PI/Annexin V staining results shown in Figure 4C. Overall, Pin1 overexpression rescued the neuronal damage induced by Pin1 knockdown, further indicating the role of Pin1 in neurons.

### 2.4. Pin1 Regulated ATRA-Induced OS and the ER in SHSY5Y Cells

**Figure 5:**
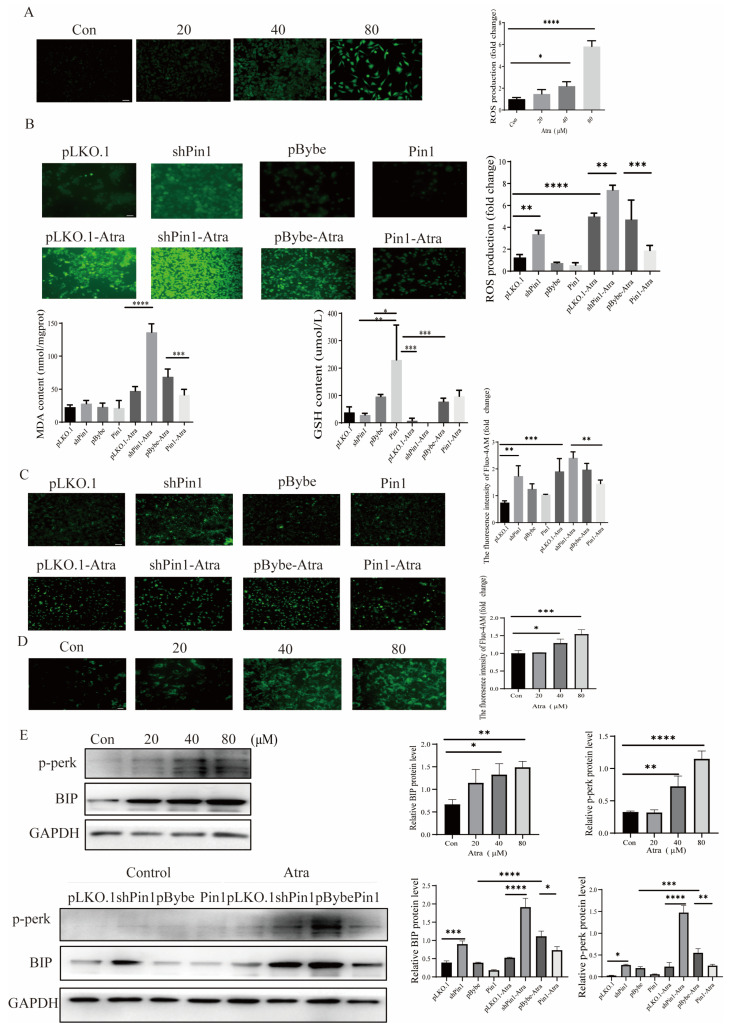
The impact of Pin1 on oxidative stress and the endoplasmic reticulum in neuronal cells. Cells were treated with varying doses of Atra or infected with control, Pin1 shRNA, or Pin1. (**A**) The ROS production levels in cells treated with different doses of Atra were determined (scale bar = 100 μm). (**B**) The ROS production levels, as well as the MDA and GSH levels, of cells infected with control, Pin1 shRNA, or Pin1 were detected (scale bar = 100 μm). (**C**) The intracellular Ca^2+^ flux was assessed in vitro (scale bar = 100 μm). (**D**) The intracellular Ca^2+^ flux in cells treated with different doses of Atra were determined (scale bar = 100 μm). (**E**) The expression levels of BIP and p-PERK were measured in vitro by WB. The data are shown as the means ± SDs from three independent experiments. * *p* < 0.05, ** *p* < 0.01, *** *p* < 0.001, and **** *p* < 0.0001 between the indicated groups.

Oxidative stress results in aberrant cell death in the developing neural tube of the embryo, leading to the formation of neural tube defects (NTDs) [18]. Thus, we determined the changes in oxidative stress indices in cell models. The findings demonstrated that there was a rise in ROS production with higher Atra concentrations, as illustrated in Figure 5A. Furthermore, in the in vitro induced cell model, the content of MDA and the production of ROS increased, and GSH decreased, and these effects were diminished following overexpression of Pin1, while knocking down Pin1 exacerbated oxidative stress in cells (Figure 5B). The ER is also critical for protein production and folding and is involved in the maintenance of cellular homeostasis [19], and activation of ER stress could lead to NTDs in the fetal stage. BIP is a key controller and prominent indicator linked to ER stress. Transmembrane ER stress sensors like PERK are recognized for their indispensable role in ER stress-induced apoptosis ref. [20], and Ca^2+^ serves as a critical determinant of cell survival and can trigger ER stress-mediated apoptosis across different contexts. ER stress can also cause Ca^2+^ overload and activate apoptosis [21]. Thus, we used BIP, p-PERK, and Ca^2+^ to investigate the effect of ER stress on cells. The addition of Atra increased the levels of BIP and p-PERK and rapidly increased the intracellular Ca^2+^ concentration; moreover, Pin1 knockdown exacerbated ER stress, while Pin1 overexpression attenuated this effect (Figure 5C,D). These data indicate that Atra exposure can induce oxidative stress and ER and that Pin1 overexpression can abrogate Atra-induced OS and ER, while Pin1 knockdown can exacerbate this damage.

### 2.5. The Knockdown of Pin1 Impacted the Proliferation of NSCs, as well as Oxidative Stress (OS) and Endoplasmic Reticulum Stress (ERs)

**Figure 6:**
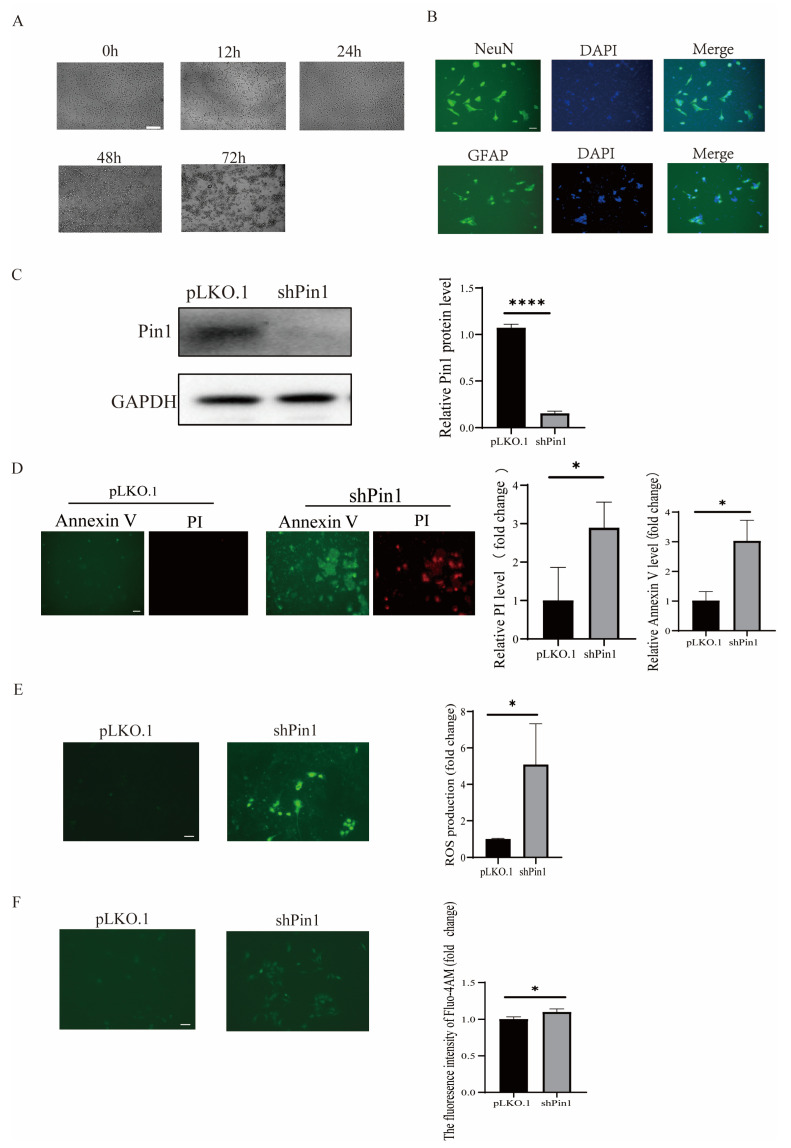
Effect of Pin1 on mouse NSCs. (**A**) NSC morphology was determined from 0 h to 72 h (scale bar = 100 μm). (**B**) NSCs were identified by immunofluorescence double staining (scale bar = 100 μm). (**C**) Western blot was utilized to identify the expression levels of Pin1 protein in NSCs. (**D**) PI/Annexin V fluorescence staining was utilized to evaluate the alterations in apoptosis levels of NSCs across various treatment groups (scale bar = 100 μm). (**E**) The changes in ROS production in NSCs induced by different treatments were detected (sale bar = 100 μm). (**F**) Changes in intracellular calcium concentration in NSCs induced by different treatment groups were examined (scale bar = 100 μm). * *p* < 0.05, and **** *p* < 0.0001 between the indicated groups.

To further elucidate the function of Pin1, a lentiviral vector with Pin1-shRNA was constructed and transduced into NSCs to knock down endogenous Pin1 expression. Additionally, a non-specific shRNA was transduced to serve as a negative control. The results indicated that shPin1 plasmid was transduced into NSCs effectively, and Pin1 expression was reduced at protein levels (Figure 6C). Then, the effect of Pin1 on NSC growth was identified by colony counting, and the results indicated that Pin1 downregulation inhibited NSC proliferation (Figure 6D). In addition, the Pin1 shRNA group exhibited increased levels of apoptosis (Figure 6E), ROS (Figure 6F), and intracellular Ca^2+^ (Figure 6F) compared to the NC shRNA group. The results were consistent with our previous results in SH-SY5Y cells and further demonstrated the function of Pin1 in the progress of neuron system development.

### 2.6. Pin1 Regulated the Notch1-Nrf2 Pathway in the Development of NTDs

The Notch and Nrf2 signaling pathways play a critical part in shaping various biological functions, such as differentiation, proliferation, and apoptosis [1]. Notch1–Nrf2 signaling crosstalk has been demonstrated, and Notch1 and Nrf2 both act as central effectors of many physiological and pathological activities related to neuronal cells [22].

**Figure 7:**
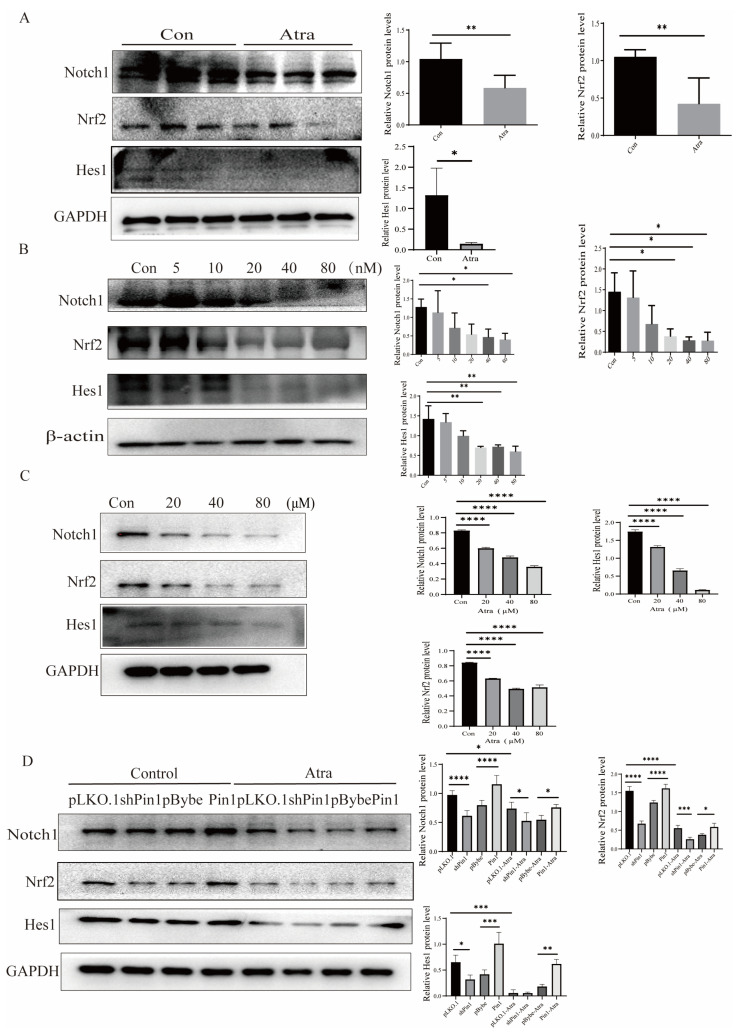
Pin1 functions via the Notch1-Nrf2 axis. (**A**) The levels of Notch1, Nrf2, and Hes1 in the mouse models were detected by WB. (**B**) The levels of Notch1, Nrf2, and Hes1 in zebrafish models were detected by WB. (**C**,**D**) The levels of Notch1, Nrf2, and Hes1 in in vitro cell models were detected by WB. The data are shown as the means ± SDs from three independent experiments. * *p* < 0.05, ** *p* < 0.01, *** *p* < 0.001, and **** *p* < 0.0001 between the indicated groups.

Therefore, we conducted WB to evaluate alterations in the Notch1-Nrf2 pathway and discovered that Notch1, Hes1, and Nrf2 were decreased not only in the mouse and zebrafish NTD models (Figure 7A,B) but also in the neuron cell model (Figure 7C). The expression of Notch1, Hes1, and Nrf2 decreased in the neural tissue of fetuses with NTDs compared to normal control fetuses (Supplementary Figure S1C), which further confirmed that Pin1 may function through the Notch1-Nrf2 axis in the development of NTDs. Thus, we speculated that Pin1 knockdown blocked the Notch1-Nrf2 signaling pathway, which enhanced neuronal damage. In contrast, Pin1 overexpression activated the Notch1-Nrf2 axis and prevented the development of NTDs.

### 2.7. Notch1-Nrf2 Inhibitors Exacerbated Atra-Induced Damage in SHSY5Y Cells

**Figure 8:**
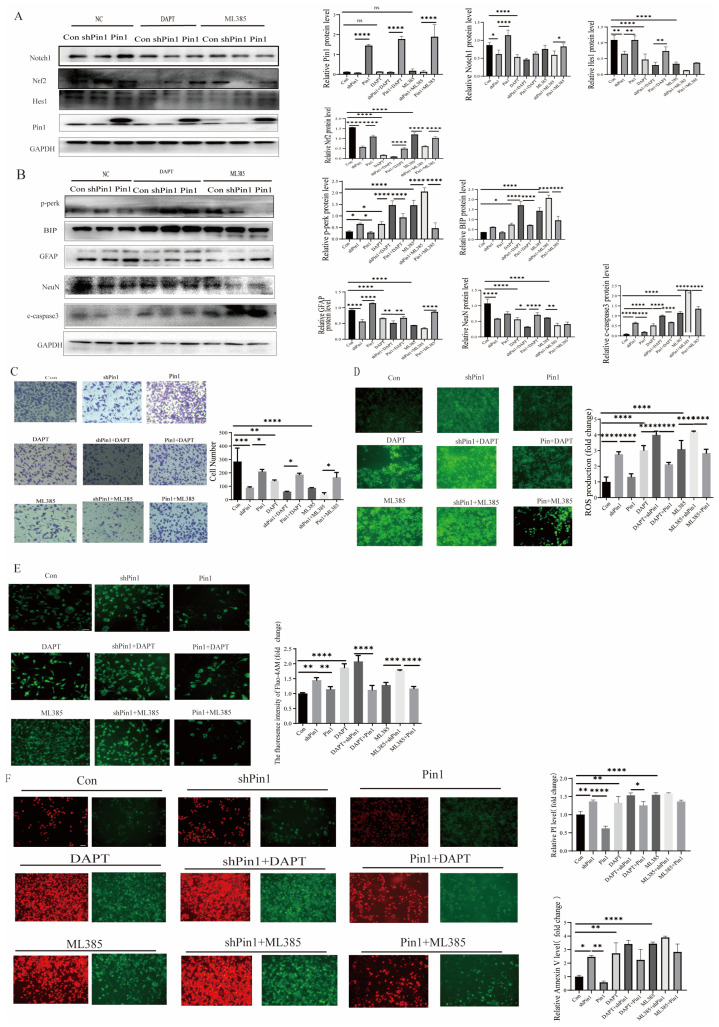
A Notch1-Nrf2 inhibitor induced neuronal cell damage. Cells were subjected to treatment with Notch1 inhibitor DAPT or Nrf2 inhibitor ML385 and infected with control, Pin1 shRNA, or Pin1. (**A**) The levels of Pin1, Notch1, Nrf2, and Hes1 in cell models were detected by WB. (**B**) The levels of BIP, p-PERK, NeuN, GFAP, and c-caspase3 were assessed using WB. (**C**) Transwell assays were employed to assess the migratory capacity of SH-SY5Y cells (scale bar = 100 μm). (**D**) The ROS production level of cells was assessed (scale bar = 100 μm). (**E**) The intracellular Ca^2+^ flux was assessed in vitro (scale bar = 100 μm). (**F**) PI and Annexin V staining were used to assess the apoptosis of SH-SY5Y cells (PI, red; Annexin V, green; scale bar = 100 μm). The data are shown as the means ± SDs from three independent experiments. * *p* < 0.05, ** *p* < 0.01, *** *p* < 0.001, **** *p* < 0.0001 and ns, no significant between the indicated groups.

**Figure 9:**
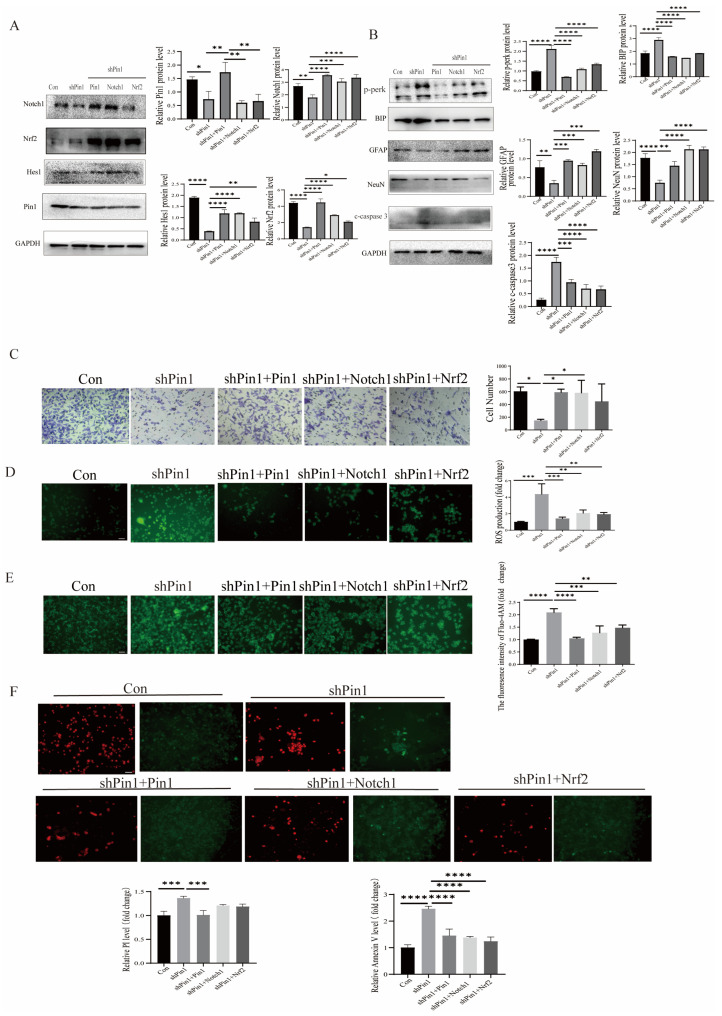
Overexpression of Pin1, Notch1, and Nrf2 effectively alleviated the neuronal damage induced by Pin1 knockdown. (**A**) The levels of Pin1, Notch1, Nrf2, and Hes1 in cell models were detected by WB. (**B**) The levels of BIP, p-PERK, NeuN, GFAP, and c-caspase3 were detected by WB. (**C**) Transwell assays were used to determine the migration ability of SH-SY5Y cells (scale bar = 100 μm). (**D**) The ROS production level of cells was assessed (scale bar = 100 μm). (**E**) The intracellular Ca^2+^ flux was assessed in vitro (scale bar = 100 μm). (**F**) PI and annexin V staining were used to assess the apoptosis of SH-SY5Y cells (PI, red; Annexin V, green; scale bar = 100 μm). The data are shown as the means ± SDs from three independent experiments. * *p* < 0.05, ** *p* < 0.01, *** *p* < 0.001, and **** *p* < 0.0001 between the indicated groups.

Notch1 inhibitor DAPT and Nrf2 inhibitor ML385 were used to further validate the expression of genes related to the Notch1-Nrf2 pathway after Pin1 was overexpressed or knocked down. The results confirmed that knocking down Pin1 may promote the development of NTDs by attenuating the activation of the Notch1-Nrf2 signaling pathway (Figure 8). Furthermore, Pin1, Notch1, and Nrf2 overexpression after Pin1 knockdown effectively alleviated neuronal damage, suggesting that the function of Pin1 may be closely associated with the Notch1-Nrf2 axis (Figure 9).

### 2.8. Pin1 Directly Interacts with Notch1-Nrf2

**Figure 10:**
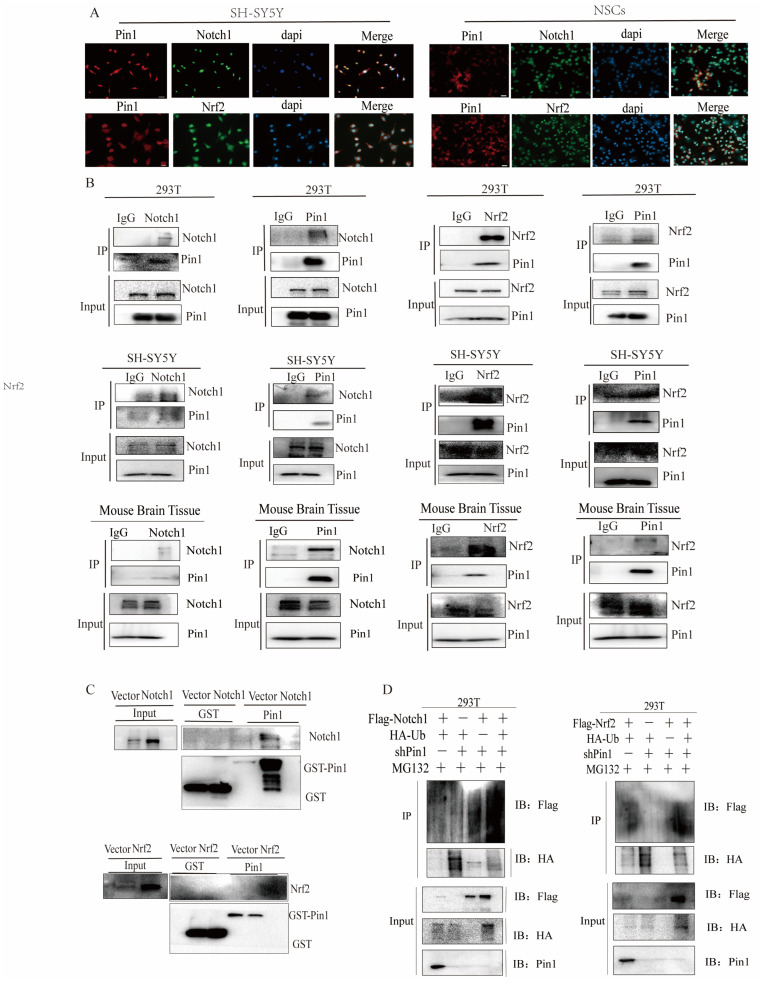
The relationship between Pin1 and the Notch1-Nrf2 axis. (**A**) Immunofluorescence assay revealed the colocalization of Pin1-Notch1 and Pin1-Nrf2 (Pin1, red; Notch1 and Nrf2, green; dapi, blue; scale bar = 100 μm). (**B**) Co-IP was used to determine the interaction between Pin1-Notch1 and Pin1-Nrf2. (**C**) GST pull-down was used to detect the direct interaction between Pin1-Notch1 and Pin1-Nrf2. (**D**) Ubiquitination assays were carried out to observe the ubiquitination level of Notch1 in the shPin1 + Flag-Notch1 + HA-ubiquitin + MG132 group and Mock + Flag-Notch1 + HA-ubiquitin + MG132 group. Ubiquitination assays were carried out to observe the ubiquitination level of Nrf2 in the shPin1 + Flag-Nrf2 + HA-ubiquitin + MG132 group and Mock + Flag-Nrf2 + HA-ubiquitin + MG132 group. The data are shown as the means ± SDs from three independent experiments.

We explored how Pin1, Nrf2, and Notch1 interact to modulate the downstream signaling pathways that cause the progression of NTDs. In SH-SY5Ycells and NSCs, we utilized immunofluorescence (IF) screening to reveal the colocalization of Pin1-Nrf2 and Pin1-Notch1 (Figure 10A). Coimmunoprecipitation (Co-IP) assays in 293T cells, SH-SY5Y cells, and mouse tissues demonstrated that Pin1 can interact with endogenous Notch1 and Nrf2 (Figure 10B), which was also supported by the results of the GST pull-down experiment (Figure 10C). What’s more, we used molecular docking to predict the possible binding sites of Pin1-Nrf2 and Pin1-Notch1 (Supplementary Figure S2).

To better understand the molecular mechanism of the Pin1-mediated Notch1-Nrf2 axis, MG132 was added to inhibit the activity of the 26S proteasome. Subsequently, using ubiquitination assays, we found that Pin1 knockdown increased the levels of ubiquitinated Notch1 and Nrf2. Thus, the above results indicated that the suppression of Pin1 expression promoted the ubiquitination of Notch1 and Nrf2 in cells and enhanced their crosstalk, which eventually accelerated the degradation of these proteins (Figure 10D).

## 3. Discussion

NTDs are among the most serious birth defects among humans, and their incidence is as high as 1 in 1000 births [23]. Failure of morphogenetic events that occur during the neurulation process could lead to serious neurological consequences or even lethality [1,24,25], placing a significant burden on both the affected individuals and society. The etiology of NTDs is multifaceted, and the mechanism of how they develop is not fully understood. The majority of reports suggest that the development of NTDs is influenced by a combination of environmental and genetic factors. In simpler terms, they are a genetic disease resulting from a combination of multiple genes and environmental influence [3,26,27].

Previous studies on NTDs have focused on the analysis of mutations and polymorphisms of key enzyme-encoding genes involved in the folate metabolism pathway. Mutations in N5, N10-methylenetetrahydrofolatereductase (MTHFR), methionine synthetase (MS), and cystathionine β-synthase(CBS), which are the three key enzymes involved in folate metabolism, can lead to homocysteine accumulation in the body and induce NTDs [28]. Therefore, supplementing with folic acid during pregnancy can effectively prevent neural tube defects (NTDs) [29,30]. Nevertheless, this mechanism does not account for the occurrence of all NTDs. Hence, it is crucial to delve deeper into the pathogenesis of NTDs. In our study, we sought to identify a target for the prevention of NTDs and showed that Pin1 is a key gene involved in Atra-induced NTDs. Moreover, the lack of Pin1 worsened Atra-induced harm both in vitro and in vivo. Finally, our study showed that Pin1 knockdown exerts its effects by blocking the Notch1-Nrf2 signaling pathway. These results indicated that Pin1 could serve as a potential target gene for the prevention of NTDs.

To determine the mechanisms involved in Atra-induced failure of neural tube closure, we used early mouse and zebrafish embryos exposed to Atra to construct NTD models. Compared with those in the control group, neural tube closure failure was detected in mouse and zebrafish embryos treated with Atra. Our results also showed that Atra exposure induced an increase in the expression of c-caspase3, an important executor of apoptosis in neural tube cells [31,32].

Pin1 plays a significant role in neurological disorders. Studies have demonstrated that, unlike in malignant tumors, Pin1 is poorly expressed in neurological disorders [33]. Research has demonstrated that Pin1 knockout mice, especially older ones, display abnormalities in cell proliferation and accumulation of Tau [34]. In addition, Pin1 is closely linked to the onset and progression of epilepsy. Studies have shown that Pin1 regulates excitatory synapses and inhibitory postsynaptic potentials in the hippocampus of Pin1 knockout mice [35,36]. Pin1 might also inhibit autophagy through the Pin1/AKT/mTOR pathway and alleviate white matter damage induced by hypoxia. Other studies have demonstrated that Pin1 might stimulate the differentiation of neural stem cells via β-catenin [13]. In line with previous studies, our findings further showed that Pin1 is decreased in Atra-induced NTD animal and cell models. Pin1 affects apoptosis, oxidative stress levels, and ER stress levels in neurons. The overexpression or knockdown of Pin1 also significantly promoted or inhibited neuron differentiation and migration in cell lines, indicating its role in neuroprotection. Overall, Pin1 plays a critical role in the progression of NTDs.

Although the phenotype of Pin1 in NTDs is well established, its specific mechanism of action have not been fully elucidated. The Notch1-Nrf2 signaling axis is closely related to the processes of cell death and differentiation. Nrf2 exhibits antioxidant properties, helps reduce nerve damage, and slows down the advancement of disease in neurons [37]. The Notch1/Hes1 signaling pathway can also facilitate the growth and maturation of neural stem cells in the hippocampus of rats with cerebral ischemia, ultimately enhancing neurological function [38]. Further studies revealed that there are several functional ARE sequences in the promoter of Notch1 and an RBPJκ binding site in the promoter of Nrf2 [22]. Furthermore, Wakabayashi et al. demonstrated that Nrf2 is a regulator of Notch1 gene activation, indicating that Nrf2 has the ability to directly influence the expression of Notch1 [39]. The findings indicated that the levels of Notch1 expression- and notch signaling-related genes were decreased in Nrf2-damaged cells. Therefore, it is conceivable that Notch1 could be the target gene of Nrf2, and Nrf2 could be the target gene of RBPJκ in the context of Notch signaling. Consequently, mutual transcription could regulate Nrf2-ARE signaling and Notch signaling, leading to a positive feedback mechanism in gene expression through Nrf2-Notch crosstalk. The Notch1-Nrf2 axis is a key molecule in nervous system development. The specific functions of the Notch1 and Nrf2 pathways in the nervous system may be closely related to apoptosis, antioxidant pathways, and neuronal differentiation. The Notch1-nrf2 axis can inhibit oxidative stress; enhance the proliferation and differentiation of neurons; prevent injury; and, thus, increase neuroprotective effects. The functions of both Notch1 and Nrf2 are closely related to Pin1 [16,17].

In the present study, we verified the effect of Pin1 on the Notch1-Nrf2 signaling pathway. Notch-Nrf2 was inhibited in the Atra-induced cellular model, while the inhibition of the Notch1-Nrf2 axis was significantly enhanced after Pin1 knockdown, and its downstream target genes exhibited corresponding effects. On the other hand, Pin1 overexpression had the opposite regulatory effect, suggesting that Pin1 knockdown may promote the progression of NTDs and exacerbate neuronal injury by regulating the Notch1-Nrf2 pathway.

To further clarify the role of the Pin1-Notch1-Nrf2 pathway and NTDs, inhibitors of Notch1 and Nrf2 were used, and Pin1, Notch1, and Nrf2 were overexpressed via Pin1 knockdown. The results validated the relationship between the Pin1-Notch1-Nrf2 axis and neuronal damage. Moreover, our research demonstrated a direct interaction between Pin1-Notch1 and Pin1-Nrf2. Our study revealed that Pin1 plays a role in neurodevelopmental processes through the Notch1-Nrf2 axis.

There are still some constraints present in our current study. CRISPER/Cas technology could be used to further verify theories related to the subject. Pin1 knockout mice and zebrafish models can be used to conduct further relevant research to deepen our understanding of the biological functions of Pin1. In addition, whether the overexpression of Pin1 in animals can delay or prevent the occurrence of NTDs is worthy of further exploration. More cases could also be collected for further experiments.

## 4. Materials and Methods

### 4.1. Chemicals and Reagents

Fetal bovine serum (FBS) was sourced from PAN Seratech (Aidenbach, Germany). Dulbecco’s modified Eagle’s medium (DMEM) and DMEM/F12 were purchased from Pricella(Wuhan, China). All-trans retinoic acid (Atra) was obtained from Sigma-Aldrich Co. (St. Louis, MO, USA). DAPT and ML385 were obtained from MedChemExpress (Shanghai, China). A Fluo-4 AM fluorescent probe was obtained from the Beyotime Institute of Biotechnology (Shanghai, China). Trypsin, penicillin, streptomycin, 4′,6-diamidino-2-phenylindole (DAPI), Triton X-100, and a bicinchoninic acid (BCA) protein assay kit were purchased from the Beyotime Institute of Biotechnology (Shanghai, China). A PI/annexin V assay kit was purchased from BD Pharmaingen (Shanghai, China). The ROS, MDA, and GSH assay kits and other biochemical assay kits were purchased from the Wuhan Elabscience Bioengineering Institute (Wuhan, China). Rabbit Notch1, rabbit NeuN, and rabbit GFAP antibodies were obtained from Cell Proteintech Group, Inc. (Wuhan, China). Rabbit Pin1, rabbit Hes1, and rabbit Nrf2 antibodies were obtained from Abcam Biotechnology (Abcam, Boston, MA, USA). Rabbit c-caspase3, rabbit BIP, and rabbit p-PERK antibodies were obtained from CST. A mouse monoclonal antibody against Pin1 was purchased from Santa Cruz Biotechnology (Santa Cruz, CA, USA). The detail could be seen in Supplementary Table S1.

### 4.2. Animals

Female C57BL/6 mice (age, 10–12 weeks; weight, 18–23 g) were mated with mature males (age, 7–8 weeks; weight, 18–25 g) overnight, and the vaginal plug was detected the following morning; embryonic day (E) 0.5 was set at noon of the day of vaginal plug detection. A total of 30 mice were utilized in the study, with the mice being randomly assigned to two groups. On day 7.5, female mice in the treatment group received intraperitoneal injections of all-trans Atra (10 mg/kg; Sigma-Aldrich; Merck KGaA, Shanghai, China) dissolved in corn oil. The other female mice in the control group received an equal amount of corn oil. Then, the pregnant mice were humanely euthanized using cervical dislocation, and the embryos were carefully dissected from the decidual tissue at E17. The mouse brains were collected to perform hematoxylin and eosin (H&E) staining, as well as immunohistological and Western blot analyses. All animal handling procedures were approved by the Animal Research Ethics Board of Fujian Medical University Laboratory Animal Center in China and complied with the institutional guidelines on the Care of Experimental Animals (GDMLAC/FL01-18-B/0).

For the zebrafish experiments, wild-type zebrafish strains from the Laboratory of Environment & Health (Vrije Universiteit—further referred to as the VU strain) and RECETOX (Masaryk University—further referred to as the MU strain) were used. At RECETOX, juvenile zebrafish (<3 months) were purchased from a local supplier and raised under standard laboratory conditions for at least 6 months prior to collection of the embryos for experiments. The fishes were fed three times a day and kept at 26 ± 1 °C in separate water tanks. At Vrije Universiteit, zebrafish of the AB strain were purchased at Ruinemans (Montfoort, The Netherlands), kept under standard conditions, fed three times a day, and kept at 26 ± 1 °C in separate water tanks.

The embryos were exposed from 4 to 72 hpf (until the measurement) without media renewal. We conducted experiments using five concentrations of Atra (5, 10, 20, 40, and 80 nM). This number of concentrations was used due to the limited amount of field samples. Most of the exposures up to 72 hpf were conducted in 6-well plates, with 30 ± 5 embryos per 2 mL per well. The embryos were placed in 6-well plates in one drop to avoid dilution of the exposure concentrations. The samples were further incubated in an incubator at a consistent temperature of 27 ± 1 °C (Thermo Scientific, Shanghai, China) to adjust the temperature to 26 ± 1 °C in a light-free water environment to prevent light-induced degradation of the retinoids.

### 4.3. Patients and Specimens

One sample of NTDs and one control were collected from the Fujian Hospital of Material and Child Health Care in 2023 to confirm the findings.

### 4.4. Cell Lines and Cell Culture

SH-SY5Y cells and human embryonic kidney-(HEK)-293T cells (purchased from the National Collection of Authenticated Cell Cultures, Shanghai, China) were cultured in DMEM/F12 or DMEM supplemented with 10% FBS, 100 units/mL penicillin, and 100 μg/mL streptomycin under a humidified atmosphere of 95% air and 5% CO_2_ at 37 °C.

### 4.5. Cell Model of NTDs

SH-SY5Y cells, a neuroblastoma cell line [31,40], are often used to induce neuronal damage and establish NTD models. SH-SY5Y cells (1 × 10^5^/well) were plated in 12-well plates, and when at 80% confluence, the SH-SY5Y cells were incubated with 50 μM Atra for 48 h, a dose chosen by MTT assay, to establish the NTD cell model.

Primary NSCs were isolated from mouse embryos at E18.5. Briefly, the brain vesicles of mouse embryos were harvested in a sterile environment, suspended as single cells, adjusted to a density of about 10^5^ cells/mL, and added to 4 mL DMEM/F12 medium (Pricella, Wuhai China) supplemented with 2% B27 (Gibco, Shanghai, China). The cells were incubated at 37 °C/5% CO_2_ and passaged every 4 days [4]. NSCs were passaged at least twice before they were used for the subsequent experiments.

### 4.6. Cell Transfection

ShPin1, PLko.1 (scrambled shRNA), Pin1, and pBybe (idle plasmid) were all acquired from GenePharma (Shanghai, China) and transfected into SH-SY5Y cells in the presence of Lipofectamine 2000 (Thermo Fisher, Waltham, MA, USA), following the manufacturer’s instructions.

### 4.7. Drug Treatment

To evaluate the cytotoxic effect of Atra, SH-SY5Y cells were stimulated with different doses of Atra (10, 20, 40, or 80 µM) for 48 h. To verify whether Pin1 contributes to Atra-induced damage via Notch1-Nrf2 signaling, SH-SY5Y cells were cultured in DMEM/F12 medium supplemented with DAPT (50 µM) or ML385 (10 µM) for 48 h after shPin1 or plko.1 transfection.

### 4.8. Cloning Assay

The cells were plated into 6-well plates at a density of 200 cells per well. The medium was changed every 3 days, and a visible colony could be seen around 14 days. At this time, the cells were washed with PBS twice, fixed with 4% paraformaldehyde for 20 min, washed again with PBS, stained with crystal violet for 5–8 min, and rinsed with water 2–3 times, then photographed.

### 4.9. Measurement of Oxidative Stress

ROS levels were detected by staining the SH-SY5Y cells with a commercial dichlorofluorescin diacetate (DCFDA) Cellular ROS Assay Kit (Abcam, Cambridge, MA, USA) following the manufacturer’s instructions. The cells were seeded and allowed to adhere in 12-well plates. After washing once with buffer (provided in the kit), SH SY5Y cells were stained with DCFDA for 45 min at 37 ℃ in the dark. After washing once with buffer, ROS generation was assessed using a fluorescence microscope. Three randomly selected fields of view were photographed.

### 4.10. Measurement of Malondialdehyde (MDA) and Glutathione (GSH) Levels

The levels of MDA, 4-HNE, and GSH were measured using a Lipid Peroxidation (MDA) Assay Kit (Sigma Aldrich, St. Louis, MO, USA) and a GSH Assay Kit (colorimetric; Abcam, Cambridge, MA, USA) following the manufacturers’ instructions.

### 4.11. Assay of Intracellular Ca^2+^ with Fluo-4 AM

SH-SY5Y cells were incubated for 24 h, then incubated for 30 min at 37 °C with 1 mM Fluo-4 AM (F14201, Beyotime, Shanghai, China) to detect alterations in intracellular Ca^2+^ levels. Intracellular Ca^2+^ levels were analyzed with a fluorescence microscope. Three randomly selected fields of view were photographed. The data were transferred to Microsoft Excel software 2016 for subsequent statistical analysis.

### 4.12. Western Blotting (WB)

WB was performed using the standard method, as reported previously [41]. An anti-GAPDH antibody and an anti-β-actin antibody were used as a loading controls to normalize the levels of other proteins. The individual bands in the Western blot were semiquantified with ImageJ 1.x software.

### 4.13. Cell Proliferation Assays

Cell proliferation was determined by 3-(4,5-dimethylthiazol-2-yl)-2,5-diphenyltetrazolium bromide (MTT) assay. The MTT assay was conducted following standard techniques, as previously reported [42].

### 4.14. Transwell Assays

For the Transwell assays, 1 × 10^5^ cells were suspended in 200 μL of serum-free medium, then seeded into the upper chamber for migration assays. The lower chamber was filled with 800 μL of whole medium. After being incubated for 24 h, the cells that had migrated through the membrane were fixed and stained with 0.1% crystal violet, then counted under a microscope.

### 4.15. PI/Annexin V Cell Apoptosis Assay

The cell apoptosis rate was detected using immunofluorescence analysis. FITC-annexin V with PI was used to assess the degree of apoptosis. The samples were incubated at room temperature for 20 min and analyzed by IF.

### 4.16. Ubiquitination Assay

Cells (transfected with scrambled shRNA and shPin1) were transiently transfected with Flag-c-Myc and HA-ubiquitin using TurboFect transfection reagent (Thermo Fisher Scientific) according to the manufacturer’s instructions for 35 h. All cells were treated with MG132 for 8 h; subsequently, the cells were lysed with IP lysis buffer and boiled at 95 °C for 10 min. The supernatant was transferred to a new tube and incubated with a Flag-tagged antibody and protein A/G agarose to purify the transfected c-Myc protein. The precipitate was analyzed by WB with anti-HA to detect the ubiquitinylated c-Myc protein.

### 4.17. Histological Analysis

Mouse and zebrafish embryos were fixed in 4% formaldehyde and embedded in paraffin. After being cut into 4 μm sections, the tissues were subjected to hematoxylin and eosin (H&E) staining. Images were obtained under a microscope (Zeiss 2.3).

### 4.18. Immunoprecipitation and GST Precipitation Assays

Immunoprecipitation assays were performed as previously described^28^. Briefly, cells were transfected for 36 h with the indicated plasmids, lysed in ice-cold immunoprecipitation buffer (50 mM Tris-HCl, pH 8.0, 150 mM NaCl, 1 mM EDTA, 1% Triton X-100, and 0.5% sodium deoxycholate) containing protease inhibitor cocktail tablets (#04693132001, Roche, Shanghai, China), and centrifuged at 14,000× *g* for 10 min. The cell lysates were then incubated with the indicated antibodies and protein A/G PLUS-Agarose (sc-2003, Santa Cruz, Shanghai, China) overnight at 4 °C, followed by washing in cold immunoprecipitation buffer. The immunocomplexes were collected and subjected to immunoblotting using the indicated primary antibodies and corresponding secondary antibodies.

GST-Pin1 fusion protein purification and GST pull-down assays. Rosetta (DE3) *Escherichia coli* was transformed with the GST-pin1 plasmid, then induced using 0.5 mM isopropyl-β-D-thiogalactopyranoside (IPTG) after the culture reached an optical density of 0.6–0.8 at 600 nm (OD600). *E. coli* extracts were prepared in PBS containing 0.5 mM MgCl_2_, 1 mM DTT, 1 mM PMSF, and protease inhibitor cocktail tablets. BeyoGold™ GST-tag Purification Resin (P2262, Beyotime, Shanghai, China) was added, and the extracts were incubated at 4 °C for 2 h. The protein-loaded beads were then incubated at 4 °C for 4 h with cells that were prepared as described above for the IP assays. The isolated proteins were washed 4–6 times in the same buffer and subjected to western blotting.

### 4.19. Statistical Analysis

Graphs were generated using GraphPad Prism 8.0 software, and the data are presented as the means ± SDs. All in vitro experiments were independently performed at least three times. Statistical analyses were performed using SPSS 22.0. Comparisons between two groups were performed using Student’s *t* test. One-way ANOVA followed by appropriate post hoc tests was used to compare more than two groups. *p* values < 0.05 were considered significant.

## 5. Conclusions

In summary, we demonstrated an abnormal decrease in Pin1 and the Notch1-Nrf2 axis in NTD patients, as well as animal and cell models. Pin1 and Notch1-Nrf2 levels were positively associated with the migration and differentiation of neurons and were negatively correlated with apoptosis, endoplasmic reticulum stress, and oxidative stress in neurons. These findings revealed potential biomarkers for the evaluation of disease processes and provided novel therapeutic targets for patients with NTDs.

## Data Availability

All data generated or analyzed during this study are available from the corresponding author upon reasonable request.

## Supplementary Materials

The following supporting information can be downloaded at: https://www.mdpi.com/article/10.3390/ijms25115588/s1.
