# Supplementary material for: Pin1 Downregulation Is Involved in Excess Retinoic Acid-Induced Failure of Neural Tube Closure
Source: Int J Mol Sci. 2024 May 21;25(11):5588. doi: 10.3390/ijms25115588 (PMC11171630; doi:10.3390/ijms25115588)
Supplement: Supplementary file 1 [file ijms-25-05588-s001.zip › Pin1 Table S1 20240503.pdf]

Table S1. Antibodies used for western blot or immunoprecipitation

Antibody	Company	Cat. No.	Dilution
Pin1 Rabbit Antibody	Abcam	ab192036-100ul	1:1000
Pin1 Mouse Antibody	Santa cruz	sC-46660	1:1000
c-caspase 3 Rabbit Antibody	CST	9664S	1:1000
BIP Rabbit Antibody	Proteintech	11587-1-AP	1:1000
p-perk Rabbit Antibody	Proteintech	29546-1-AP	1:1000
GAPDH mouse Antibody	Trans Gene	HC301-01	1:1000
HRP Goat Anti-Rabbit IgG	Beyotime	511203	1:5000
HRP Goat Anti-Mouse IgG	Beyotime	511103	1:5000
NeuN Rabbit Antibody	Proteintech	26975-1-AP	1:1000
GFAP Rabbit Antibody	Proteintech	16825-1-AP	1:1000
Nrf2 Rabbit Antibody	Abcam	12721S	1:1000
Notch1 Rabbit Antibody	Proteintech	20062-2-AP	1:1000
Hes1 Rabbit Antibody	CST	11988S	1:1000
