# Supplementary material for: Pin1 Downregulation Is Involved in Excess Retinoic Acid-Induced Failure of Neural Tube Closure
Source: Int J Mol Sci. 2024 May 21;25(11):5588. doi: 10.3390/ijms25115588 (PMC11171630; doi:10.3390/ijms25115588)
Supplement: Supplementary file 1 [file ijms-25-05588-s001.zip › Supplementary Figure with Figure legend.pdf]

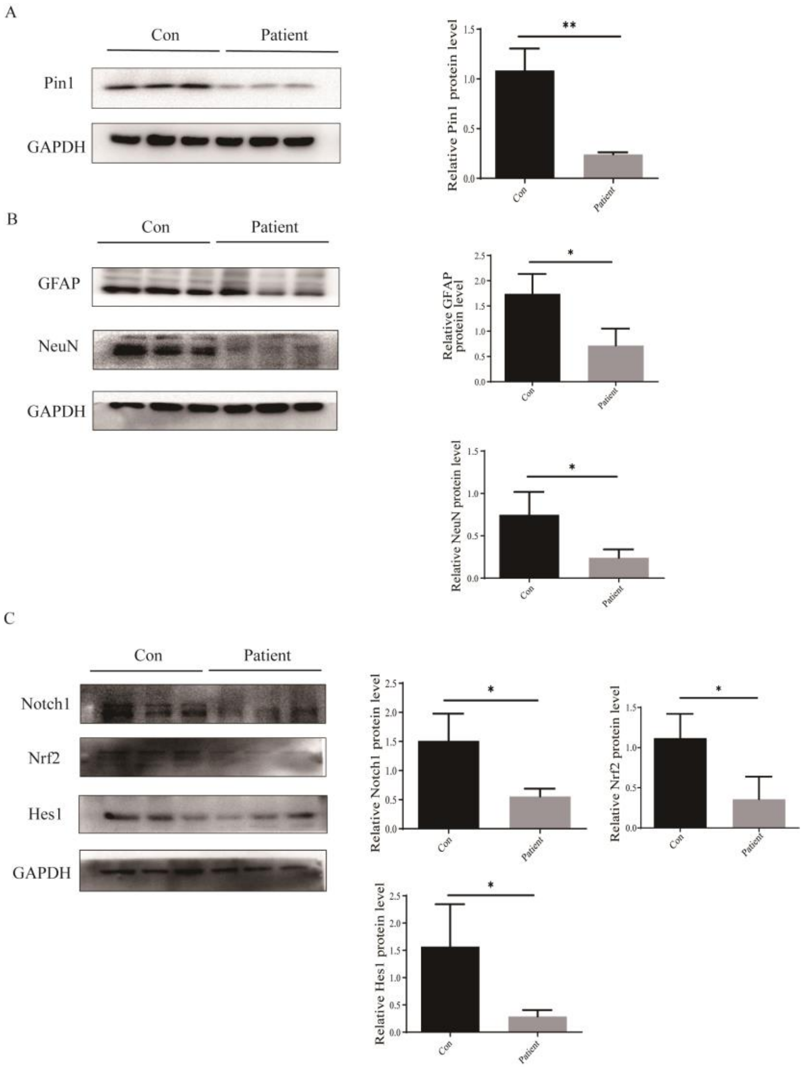

Supplementary Figure S1

Supplementary Figure S1 The function of Pin1 in clinical cases. (A) Western Blot was used to detect the expression of Pin1 in the brain tissues of control and Neural tube defect patients. (B) Western Blot analysis of the expression of GFAP and NEUN in control and Neural tube defect brain tissues. (C) Western Blot analysis of Notch1, Hes1, and Nrf2 expression in control and Neural tube defect brain tissues. *P < 0.05, **P < 0.01between the indicated groups.

A

Rank	1	2	3	4	5	6	7	8	9	10
Docking Score	-279.83	-252.21	-239.01	-238.61	-238.6	-236.95	-236.79	-236.71	-235.7	-234.35
Confidence Score	0.9306	0.8854	0.8557	0.8547	0.8547	0.8506	0.8502	0.8499	0.8474	0.8438

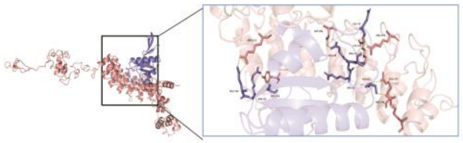

Pin1_3tc5:Blue
Notch1： Red

Pin1	Notch 1	Interaction
GLU163	ARG213	Hydrogen bond
ARG54	TYR225	Hydrogen bond
SER126	TYR225	Hydrogen bond
ARG69	ASP76	Hydrogen bond
PRO70	LYS316	Hydrogen bond
GLU76	LYS316,ARG[illegible]47	Hydrogen bond
SER65	ARG357,ARG91	Hydrogen bond
ARG68	LYS316	Hydrogen bond

B

Rank	1	2	3	4	5	6	7	8	9	10
Docking Score	-249.44	-233.19	-230.92	-227.2	-226.03	-220.94	-219.53	-219.37	-219.24	-215.14
Confidence Score	0.8796	0.8407	0.8346	0.824	0.8206	0.8052	0.8007	0.8002	0.7998	0.7863
Ligand rmsd (Å)	139.11	129.45	121.08	168.78	169.36	143.3	221.49	125.41	127.42	222.47

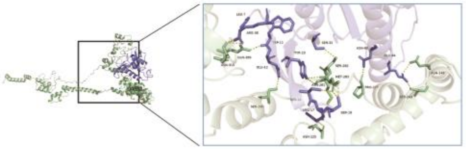

Pin1_3tc5： Blue
NRF2： Green

Pin1	NRF2	Interaction
LEU7	ASN315	Hydrogen bond
ARG36	ASN315	Hydrogen bond
TRY11	GLN389	Hydrogen bond
GLU12	SER290	Hydrogen bond
TYR23	ASN282, MET283	Hydrogen bond
SER32	SER282	Hydrogen bond
SERE16	ASN281	Hydrogen bond
ARG17	ASN225	Hydrogen bond
SER18	ASN281	Hydrogen bond
ASN90	PRO277	Hydrogen bond
GLU84	ASN248, SER243	Hydrogen bond

Supplementary Figure S2

Supplementary Figure S2 Molecular docking predicted the interaction and possible binding sites of Pin1 with Notch1 and Pin1 with Nrf2.
